# The FJQR Has Synergistic Effect with Fluoropyrimidine in the Maintenance Treatment for HER-2 Negative Gastric Cancer

**DOI:** 10.2174/1574892818666230522161742

**Published:** 2024-12-04

**Authors:** Fanming Kong, Lu Zhao, Na Wang, Dou Zhang, Ziwei Wang, Qingyun Mei, Yongchao Yu, Yingjie Jia

**Affiliations:** 1 Department of Oncology, First Teaching Hospital of Tianjin University of Traditional Chinese Medicine, Nankai District, 300193, Tianjin, China;; 2 National Clinical Research Center for Chinese Medicine Acupuncture and Moxibustion, 300193, Tianjin, China

**Keywords:** Gastric cancer, maintenance therapy, traditional chinese medicine, fluoropyrimidine, FJQR, HER-2

## Abstract

**Introduction:**

Maintenance therapy aimed to strengthen the first-line chemotherapy and improve quality of life is needed for gastric cancer (GC). Currently, many clinical studies have confirmed the important role of fluoropyrimidine in the maintenance stage. Our team has created patented prescriptions “Fuzheng jiedu Quyu Method” recipe (FJQR), which was considered as an adjuvant therapeutic scheme (reduce toxicity and increase the efficacy of chemotherapy). This study aimed to evaluate the efficacy and safety of FJQR combined with fluoropyrimidine as a maintenance treatment in HER-2 negative GC patients.

**Methods:**

We performed the analysis of 129 patients with HER-2 negative GC who entered the maintenance stage in our hospital and Tianjin Cancer Hospital between January 2018 and December 2020. Out of the 129 eligible patients, 64 were categorized into the maintenance treatment group with FJQR+fluoropyrimidine, and 65 patients were assigned to the control group if they received fluoropyrimidine alone. Capecitabine was orally 1000mg/m^2^, Qd, half an hour after meals, and FGQR was 15g Bid after capecitabine. The primary endpoint was progression-free survival (PFS). The secondary endpoints were overall survival (OS), overall remission rate (ORR), quality of Life (QOL), TCM syndrome and safety.

**Results:**

The mPFS in the treatment group was significantly prolonged compared with the control group (6.3 *vs*. 5.0 months, *p* = 0.03), while the mOS was not substantially improved (11.4 *vs*. 10.5 months, *p* = 0.38). Gastrointestinal symptoms and pain became better in the treatment group. The number of distant metastatic organs, first-line chemotherapy cycles, and lymph node metastasis were independent risk predictors for PFS. Blood stasis syndrome may be the protective factor. In terms of safety, treatment-related adverse events (AEs) in the treatment group were relatively lighter, and the incidence of grade III-IV AEs could be significantly reduced.

**Conclusion:**

FJQR and fluoropyrimidine have synergistic effects as maintenance treatment in HER-2 negative GC, with good efficacy and safety.

## 1 INTRODUCTION

Advanced gastric cancer (GC) is the fifth common neoplasm characterized by a high rapid growth rate and is the major cause of cancer-related death worldwide, particularly in China [1, 2]. Human epidermal growth factor receptor 2 (HER-2) negative GC represented the majority of the cases (80-88%) [3]. The trastuzumab-based regime was confirmed as the standard first-line treatment for HER-2-positive GC patients [4, 5]. In contrast, over the past decade, no new drugs have been approved for advanced HER-2 negative GC patients. The 5-year survival rate was still poor (<5%) [6]. In recent years, platinum combined with fluoropyrimidine remained the basic first-line treatment. It was used until disease progression or uncontrollable adverse events (AEs) occurred in this part of patients, yet the ideal duration of first-line chemotherapy has not been confirmed [7-9]. However, the cumulative toxicity and decreased performance status forced this part of patients do not tolerate a full course of chemotherapy. Besides, the second-line treatment only benefited 40% of HER-2 negative GC patients [3, 10]. Therefore, consolidating the efficacy of first-line treatment and delaying tumor progression was significant.

In recent years, maintenance therapy has been successfully used in multiple cancers to delay disease progression and improve quality of life. The earliest successful application of maintenance therapy (pemetrexed) was in advanced non-small cell lung cancer [11]. After that, flu-uracil also achieved satisfactory results when maintained in colorectal cancer [12, 13]. As for GC, many trials were designed with fluoropyrimidines such as capecitabine tegafur and tigio as maintenance therapy in HER-2 negative GC, and the reported mPFS and mOS seemed longer [6, 14, 15]. Nevertheless, the accumulated toxicity and insupportableness side effect result in disadvantageous survival benefits, which limited its application for a long time. With the emergence of more innovative drugs, strategies of maintenance therapy in advanced HER-2 negative GC are becoming more selective.

Traditional Chinese Medicine (TCM) has the characteristics of multiple targets, slight side effects and excellent therapeutic outcomes. TCM prescription, Chinese patent medicine and TCM monomer compound showed good anti-tumor efficacy. Now many studies have demonstrated that applying TCM can significantly reduce the adverse reactions caused by chemotherapy, prevent recurrence and metastasis, enhance patient quality of life, and finally prolong survival. Li *et al*. proved herbal treatment decoction with Astragalus mongholicus and Semen cuscutae affected leukocyte and QoL scores in patients with chemotherapy of advanced GC [16]. At the same time, Qi *et al*. indicated that intravenous artesunate was 18 mg/kg, which the treatment of GC was well tolerated and diminishing the side effects [17]. Similarly, Hong *et al*. showed XiangShaLiuJunZi decoction combined with S-1 performs well in stage III or IV GC patients. The treatment group had better PFS and improved KPS scores [18]. However, none of the above trials have conducted the subtypes of GC, so the beneficiary population was not precise and clear. Most of them were case reports, which have no *in vitro* experiments. The “Fuzheng jiedu Quyu Method” recipe (FJQR) was the prescription of the First Teaching Hospital of Tianjin University of Traditional Chinese Medicine. It has an effective anti-tumor effect in clinical applications, and preliminary studies have found that FJQR combined with chemotherapy can reduce toxicity and increase efficiency.

There is a lack of evaluation on the efficacy and safety of FJQR combined with fluoropyrimidine as maintenance therapy for advanced HER-2 negative GC. This study will provide the true clinical value of the FJQR compound combined with oral fluorouracil drugs in the maintenance treatment of advanced HER-2 negative GC patients.

## 2 MATERIALS AND METHODS

Our study protocol was approved by the Institutional Review Board of the First Teaching Hospital of Tianjin University of Traditional Chinese Medicine, Tianjin, China (TYLL 2022[Y]004).

### 2.1 Study Population

According to the sample size calculation method, using a bilateral test, α = 0.05, test efficiency 1-β = 0.90 (β = 0.10), the estimated number of cases in each group was 59, and a total of 118 patients were in the two groups. Considering the loss of follow-up rate of 10% to 20% and the specific stratification analysis in the later stage, 156 patients were included in this study.

From January 2018 to December 2020, data from 156 advanced HER-2 negative GC patients without progression after induction chemotherapy and then treated with fluoropyrimidine monotherapy or FJQR+fluoropyrimidine combination therapy as maintenance treatment in 2 institutions across China were retrospectively collected in this study, namely, First Teaching Hospital of Tianjin University of Traditional Chinese Medicine and Tianjin Cancer Hospital. Finally, according to the eligibility criteria, 129 patients were eventually included.

#### 2.1.1 Eligibility Criteria

Patients aged from 18 to 75 years old; pathology stage IV AGC; HER-2 negative (Immunohistochemistry and FISH); at least one measurable lesion can be detected; Eastern Cooperative Oncology Group (ECOG) performance status (PS) score: 0-2; achieve to CR, PR or SD after first-line chemotherapy and entered maintenance stage; diagnosed as qi deficiency, toxin, and blood stasis; complete clinical pathology data were available, patients and their families were willing to cooperate with the follow-up.

#### 2.1.2 Exclusion Criteria

Combined with other malignancies; had an uncontrolled or symptomatic systemic disease (*e.g*., hypertension, coronary heart disease, arrhythmias, cardiac insufficiency active hepatitis B, AIDS, or Alzheimer’s disease); maintenance therapy used radiotherapy, chemotherapy, immunotherapy, targeted therapy, and other treatments; patients who could not take oral Chinese medicine or with frequent vomiting; absence of the raw data affected the test.

### 2.2 Study Design and Therapeutic Regime

An information Questionnaire was drawn up according to the latest clinical research worldwide. Baseline data, including relevant baseline tests, were collected according to the clinical observation forms before enrollment. According to different interventions in the maintenance stage, a total of 129 patients were classified into Fluoropyrimidine plus FJQR (Treatment group, n = 64) and Fluoropyrimidine (Control group, n = 65) groups. FJQR was provided and prepared by the Pharmacy of the First Teaching Hospital of Tianjin University of Traditional Chinese Medicine (Z20160002), a 9g Bid, 28 days as a cycle of administration. The dose of capecitabine (GYZ ZI H20073024, Shanghai Roche Pharmaceuticals Ltd.) was 1000mg/m^2^, Qd, half an hour after meals (the time of daily medication was as same as possible) for 2 consecutive weeks. The 21 days treatment cycle included 1 week without medication. The dose suspension and reduction were allowed when hematological toxicity reached level 3 or non-hematological toxicity reached level 2. For non-hematologic toxicity, controllable nausea, vomiting, hair loss, fever with an established cause (*e.g*., infection or tumor), and grade 3 or 4 alkaline phosphatase elevation were treated with active symptomatic management and without dose suspension or dose downgrading. All patients continued to be assessed for safety within 28 days at the end of the last dose. After 28 days of safe follow-up to the end of death or loss of follow-up, contact patients at least once every month to collect survival information after treatment

### 2.3 Efficacy Assessment

Baseline data, including relevant baseline tests, were collected according to the clinical observation forms before enrollment. Before starting treatment, patients received the following tests: CT, MRI, 12-lead electrocardiogram examination, blood count, liver and kidney function, blood pressure monitoring, tumor markers, routine urine tests, electrocardiogram, KPS, neurological examination, and physical examination. The above tests were also implemented every 14 days during the first two maintenance cycles, thereafter, every two months. CT or MRI was performed at the end of each cycle to evaluate the objective efficacy. The radiology specialist and attending doctor reviewed the imaging data.

PFS was defined as the time from initial maintenance treatment to either progression or death. The results were censored if the patient had no known disease progression at the last follow-up. OS was defined as the time from initial maintenance treatment to death from any cause. The results were censored if the patient was alive at the last follow-up. ORR included CR and PR. The Quality of Life (QOL) scale indicated the symptom; the total score is 100 points. The following dimensions were involved: dysphagia, body pain, reflux symptoms, dietary restriction, anxiety, psychology, holistic health and so on. A higher score represents a better quality of life. The TCM syndrome score is based on the “Guiding Principles for Clinical Research of New Chinese Medicines.” Stomachache, abdominal distention, acid reflux, poor appetite, belching, vomiting, fatigue, constipation and loose stool were included.

### 2.4 Safety Assessment

This study evaluated all complications developed using National Oncology Institute General Poison Side Standard (National Cancer Institute Common Toxicity Criteria 3.0, NCT-CTC3.0). In our study, grades II-III were defined as moderate adverse events and grade V was defined as death. The major adverse events included leucopenia, anemia, thrombocytopenia, nausea, pigmentation, hand-foot syndrome and oral mucositis.

### 2.5 Statistical Analysis

The test results were mainly described by SPSS 25.0 statistical software for Windows. The baseline characteristics of the two groups were compared by Fisher’s exact test and T-test. ORR and DCR were calculated by logistic regression analysis. The measurement data listed the mean, standard deviation, and median, while the counting and grade data listed the frequency (constituent ratio), rate, and confidence interval. Kaplan-Meier drew the survival curve, and Cox proportional hazards analysis evaluated risk factors on PFS. *p*-values less than 0.05 were statistically significant.

## 3 RESULTS

### 3.1 Patients Characteristics

**Fig. (1):**
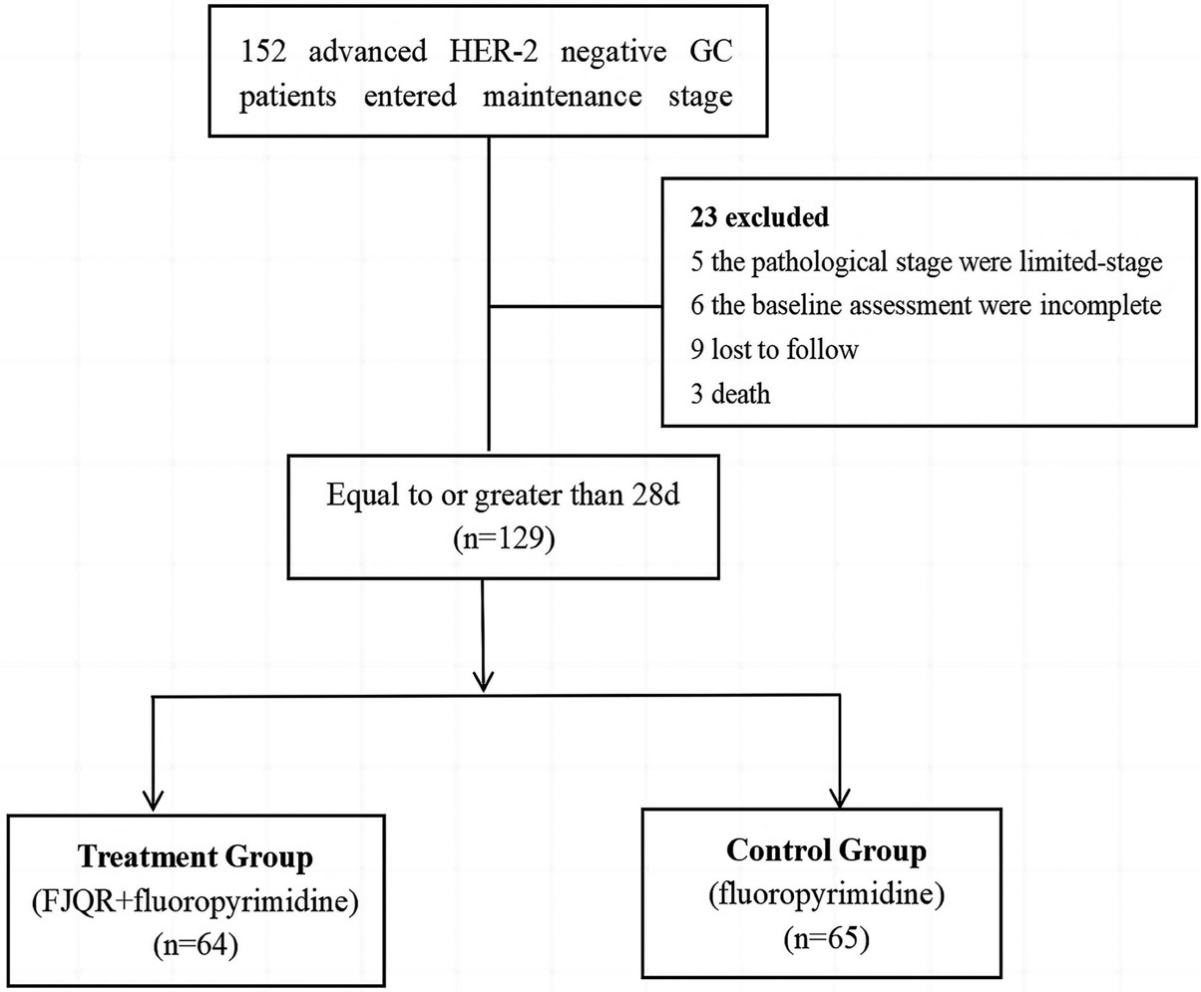
Experimental STUDY flow chart.

152 advanced HER-2 negative GC patients who completed the first-line chemotherapy without progression were screened. Among them, 23 were excluded: 5, the pathological were limited-stage, 6, the incomplete baseline assessment, 9 were lost to follow, and 3 died. Finally, 129 consecutive patients with histologically confirmed stage IV HER-2 negative GC were identified at our hospital and Tianjin Cancer Hospital between January 2018 and December 2020. Of them, 64 cases received FJQR+fluoropyrimidine, and 65 cases accepted fluoropyrimidine monotherapy in the maintenance treatment (Fig. **1**). Table **1** showed that the two groups did not significantly differ in terms of gender, age, differentiation, ECOG performance status, drinking history, surgical condition, first-line chemotherapy regimen, first-line chemotherapy cycle, response to chemotherapy, lymph node metastasis and the number of metastatic sites (*p* >0.05), so they were comparable.

### 3.2 Survival Time

**Fig. (3):**
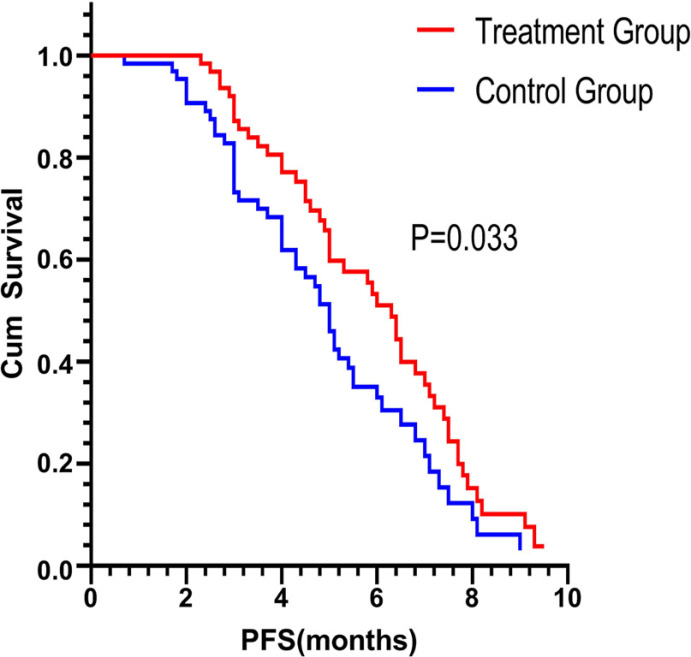
The mPFS in the treatment group was significantly prolonged compared with the control group (6.3 *vs*. 5.0 months).

The clinical efficacy evaluation showed that the treatment group compared to the control group had better outcomes (CR: 0%, PR: 9.4% *vs.* 6.2%, SD: 67.2% *vs.* 60%). Therefore, the ORR was 9.4% *vs* 6.2% (*p* = 0.048). The DCR was 76.6% *vs.* 66.2% (*p* = 0.382). The waterfall plot of the treatment group response is shown in Fig. (**2**). The mPFS was 6.3 months for patients receiving FJQR plus fluoropyrimidine *vs* 5.0 months for patients having fluoropyrimidine monotherapy in the maintenance stage (*p* = 0.033 <0.05). (Fig. **3**). The overall survival was 11.4 months *vs.* 10.5 months in the treatment and control groups, respectively (*p* = 0.384 >0.05, Fig. (**4**). These results were in line with the previous studies.

### 3.3 Independent Risk Factors for PFS

We included gender, age, ECOG status, previous surgery, lymph node metastasis, the cycle of treatment, and No. of metastatic organs for analysis. Single-variate for PFS showed the number of metastatic sites, first-line chemotherapy cycles, TCM syndrome type, and lymph node metastasis were statistically significant (*p* <0.05, Table **2**). Then, multivariate analysis demonstrated that lymph node metastasis, number of metastatic sites and number of first-line chemotherapy cycles were independent factors of PFS (*p* <0.05, Table **3**).

### 3.4 TCM Symptoms

**Fig. (4):**
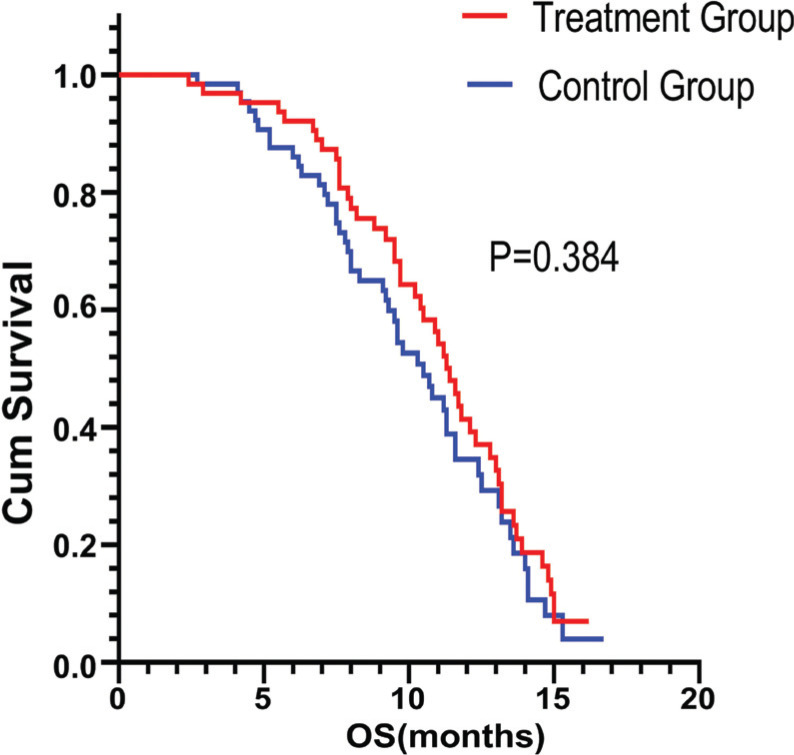
The mOS was not substantially improved (11.4 *vs*. 10.5 months).

The questionnaire on TCM symptoms was based on the TCM Diagnosis and Treatment Scheme for Gastric Cancer issued by the Department of Medical Administration in the National Administration of Traditional Chinese Medicine in 2011. At the start of the analysis, we compared the TCM symptoms of the two groups, and the difference was not statistically significant (*p* >0.05). After all cycles of first-line chemotherapy, most patients presented with abdominal distention, poor appetite, fatigue, stomachache, and vomiting, which indicated that chemotherapy would damage the energy and produce many clinical symptoms that make people uncomfortable. Using Chi-square test analysis after maintenance treatment, the chronic symptoms of fatigue, abdominal distention, and vomiting were improved in the treatment group (*p* <0.05, Fig. **4**).

### 3.5 Quality of Life

The quality of life of the total 129 patients was assessed by two clinicians using the Quality of Life scale. The SS analysis showed that the two groups were comparable (*p* >0.05). After maintenance treatment, the results indicated that the stomachache and reflux symptoms improved more in the treatment group than in the control group (*p* <0.05, Table **4**).

### 3.6 Safety

Most AEs in the treatment group were in grades I-II, relieved after symptomatic treatment, and patients could tolerate the subsequent treatment. The most common AEs were leucopenia, hepatorenal dysfunction, and nausea/vomiting. The comparison of AEs between the two groups showed no statistically significant occurrence of any grade of AEs. Table **5** for details. Notably, the incidence of grade III-IV AEs in the FJQR combined with the fluoropyrimidine group was significantly reduced.

## 4 DISCUSSION

A patent was awarded to a TCM formula FJQR for treating advanced GC. It consists of Astragalus (Huang Qi), Pseudostellariae radix (Taizi Shen), Spica Prunellae (Xiaku Cao), Curcuma (Jiang Huang), Curcumae radix (Yu Jin), Herba Oldenlandiae (Baihua Sheshe Cao). Its main chemical components are Astragalus polysaccharides, Radix Prunella, Prunella vulgaris saponins, *etc*. In clinical practice, our team found FJQR has a synergistic effect with chemotherapy. Hu *et al*. proved FJQR, combined with paclitaxel, can improve short-term outcomes and reduce any grade of AEs in NSCLC. The efficiency rate was 53.3% *vs*. 33.3% in the FJQR+paclitaxel and cisplatin+paclitaxel groups. Meanwhile, Zhang *et al*. demonstrated FJQR inhibited the expression of VEGF, thus alleviating the AEs of the hematopoietic system and improving the quality of life. Recently, Yang *et al*. found FJQR had a synergistic effect with 5-FU in the corresponding concentration, and when the drug effect<0.7, the CI<1 [19, 20]. And FJQR inhibited MKN45 then induced apoptosis after being combined with 5-FU. Based on clinical efficacy, through basic experimental research, it is preliminarily confirmed that FJQR prescription inhibits the proliferation, migration, and molecular signaling pathways by regulating TGF-β/Smad3/MMP-9, Akt1-mTOR, Beclin1-YAP1 and inhibiting the expression of Survivin gene then promoted A549 apoptosis [21, 22].

Maintenance therapy was aimed at controlling disease after initial treatment. Based on the available studies, two commonly used maintenance types were confirmed: continuation maintenance (small doses of the initial drugs) *vs*. switch maintenance (other low-toxicity drugs for sustained treatment) [23]. This strategy was to delay the recurrence or deterioration of symptoms and to prolong PFS as much as possible before tumor progression. Nowadays, fluoropyrimidines such as capecitabine, tegafur and tigio are frequently used as maintenance therapy in HER-2 negative GC patients [24]. Nevertheless, the accumulated toxicity and insupportableness side effects resulted in a disadvantageous survival benefit, which was unsuitable for a long time. Therefore, the optimal maintenance regimen is still absent.

This retrospective study found that maintenance with FJQR significantly improved PFS compared to chemotherapy alone in the real world (6.3 months *vs.* 5.0 months). Because the PFS reflects the direct effect of maintenance therapy and is not affected by the cross-line and post-line treatment, we selected PFS as the primary endpoint. Noteworthy, our results were more optimistic compared with other studies. A prospective clinical study showed that maintenance with 5-fluorouracil (5-FU) after the FOLFOX-4 regimen could improve the median PFS (5.9 months) and mOS (9.6 months) in advanced HER-2 negative GC patients [25]. Moreover, a retrospective study using avelumab maintenance for HER-2 negative GC patients reported that mPFS was 2.8 months and mOS were 11.1 months [26]. Another clinical phase III trial used ramucirumab in combination with paclitaxel maintenance in stage IIIB-IV HER-2 negative GC patients; the results showed that the PFS was not reached [27]. The above-mentioned trials did not report substantial PFS, but the findings of this study presented a considerably increased survival time. Based on our previous cytology experiments, FJQR, in combination with “Protein Knockout,” can significantly inhibit GC cells from growing, enhance the efficacy of 5-FU, and reverse drug resistance [28, 29]. Therefore, improving PFS may be related to the anti-tumor synergistic effect of FJQR and fluoropyrimidine. However, the OS was not statistically prolonged. It agreed with the available reports, probably because non-mobility therapy was influenced from the beginning to the end of chronic diseases.

We analyzed the effects of different factors on PFS, including gender, age, differentiation, previous surgery, the cycle of treatment and chemotherapy regimen, *etc*. The results showed that the number of metastatic sites, first-line chemotherapy cycles, TCM syndrome type, and lymph node metastasis significantly affected PFS. Furthermore, we used multivariate Cox regression to analyze the independent risk predictors of PFS and found that the lymph node metastasis, number of metastatic sites and number of first-line chemotherapy cycles were independent risk factors for PFS. TCM was characterized by organic wholeness and treatment based on syndrome differentiation. So, although blood stasis syndrome was not the independent protective factor for PFS, we found that this type could potentially reduce the risk of disease progression by analyzing the subgroup.

Patients with GC for more than one month have a high incidence of chronic symptoms of stomachache, reflux symptoms, fatigue, abdominal distention and vomiting. Our results showed that the stomachache, reflux symptom, fatigue and abdominal distention had a higher improvement in the treatment group, indicating that FJQR can improve chronic clinical symptoms such as fatigue, reflux symptom, stomachache and so on. The possible mechanism of action FJQR can block DA and 5-HT receptors. In our study, 41 patients would tolerate the second-line or third-line treatment after the chronic symptoms improved, compared with 35 patients in the control group, which indicated that FJQR increased the probability of HER-2 negative GC patients receiving post-line treatment.

There was no statistically significant occurrence of any grade of AEs between the two groups. Also, the adverse reactions were usually mild, mainly in degrees I and II. Notably, the incidence of grade III-IV AEs in the XYG combined with the apatinib group was significantly reduced. This was because FJQR contained excellent proteins and amino acids, which can remove the active acid and strengthen immunity. Furthermore, FJQR balanced the expression of erythropoietin, thrombopoietin, and granulocyte-macrophage colony-stimulating factor (GM-CSF) in the marrow microenvironment. Promote hematopoietic cells from G0/G1 to G2/M and S. Thus improving myelogram and hemogram [30]. It was suggested that the combined application of FJQR was safe and had no notable clinical toxicity; even more, it can relieve patients' discomfort.

The patients selected in this study were relatively standard and representative, which can provide clinical evidence for apatinib as maintenance therapy in advanced HER-2 negative GC patients. Of course, several limitations should be acknowledged. On the one hand, our results were obtained from a retrospective study over a long period, and a small number of cases will have some interference. On the other hand, corresponding clinical data was absent, so the in-depth molecular and specific mechanisms analysis can not be ongoing. Furthermore, more *in vitro* and *in vivo* studies are needed to support these findings. I look forward to seeing more results in the near future.

## CONCLUSION

Acknowledging our limitations, we can conclude that FJQR plus fluoropyrimidine was an efficient and safe maintenance treatment in HER-2 negative GC patients. And FJQR can reduce toxicity and increase the efficiency of fluoropyrimidine. This maintenance regime was supposed to be confirmed in further prospective randomized research.

## CURRENT& FUTURE DEVELOPMENTS

FJQR is a TCM preparation of the First Teaching Hospital of Tianjin University of Traditional Chinese Medicine. It can inhibit tumor growth, prevent migration, and improve immunity, making a better prognosis. Our work demonstrated that JPJDF can slow the progression and reduce the side effects of chemotherapy in the maintenance stage. This regime is relatively effective and safe, which was supposed to be confirmed in further research. In the future, we tried to provide, through a more comprehensive application of FJQR, a new option for treating HER-2-negative gastric cancer and other advanced cancers.

## Figures and Tables

**Fig. (2):**
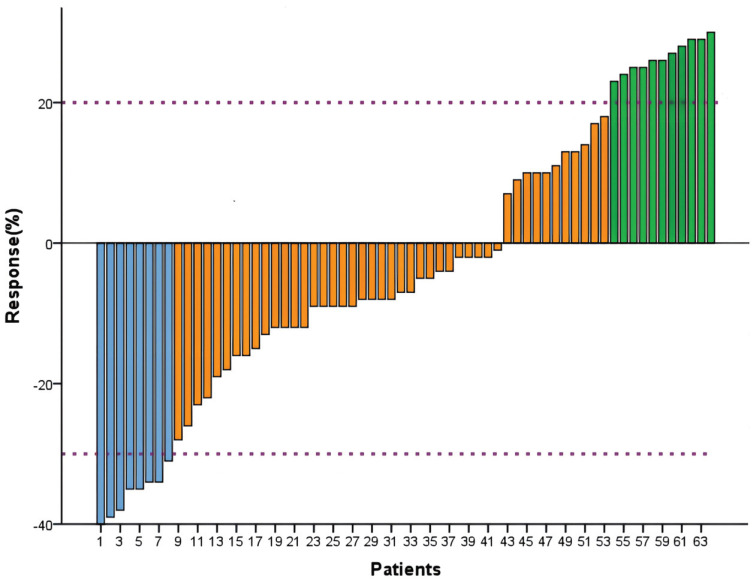
The waterfall plot of the treatment group response.

**Table 1:** Basic characteristics of patients.

**Characteristics**	**Groups**		***p*-value**	**X^2^- value**
	**Treatment Group (n = 64)**	**Control Group (n = 65)**		
**Gender**				
Male Female	44(68.8%) 20(31.2%)	48(73.8%) 17(26.2%)	0.522	0.409
**Age**				
≤45 46-70 >70	5(7.8%) 46(71.9%) 13(20.3%)	4(6.1%) 44(67.7%) 17(26.2%)	0.761	0.727
**Differentiation**				
High Middle Low	0(0%) 8(12.5%) 8(12.5%)	0(0%) 9(14.0%) 56(86.2%)	0.832	0.707
**ECOG Performance Status**				
0-1 2	49(76.6%) 15(34.4%)	43(66.2%) 22(33.8%)	0.077	3.126
**Drinking History**				
Yes No	39(60.1%) 25(29.1%)	34(52.3%) 31(47.7%)	2.508	0.665
**Surgical Condition**				
Yes No	24(37.5%) 40(62.5%)	31(47.7%) 34(52.3%)	0.242	1.370
**First-line Chemotherapy Regime**				
Two drugs Three drugs	55(85.9%)	54(83.1%)	0.654	0.201
	9(14.1%)	11(16.9%)		
**First-line Chemotherapy Cycle**				
>4 4-6 >6	16(25%)	14(21.5%)	0.798	0.624
	31(48.4%)	36(55.4%)		
	17(26.6%)	15(23.1%)		
**No. of Metastatic Organs**				
0 1-2 >2	9(14.1%) 16(25%) 39(60.9%)	12(18.5%) 18(27.7%) 35(53.8%)	0.686	0.755
**Lymph Node Metastasis**				
Present Absent	39(60.9%) 25(39.1%)	41(63.1%) 24(36.9%)	0.054	7.605

**Note:** CR means complete response, PR means partial response, SD means stable disease.

**Table 2:** Uni-variate analysis of PFS in treatment groups.

**Characteristic**	**Cases**	**mPFS (Months)**	**χ^2^-Value**	**95%CI**	**χ^2^-Value**
**Gender**					
Male	44	6.4	0.137	5.04-7.76	0.712
Female	20	5.8	-	3.69-7.64	-
**Age**					
≤45yrs	5	5.8	-	3.01-8.59	-
46-70yrs	46	6.0	1.192	4.10-7.90	0.551
>70yrs	13	6.4	-	4.44-8.36	-
**Differentiation**					
YES	10	8.2	24,248	6.81-9.59	<0.01
NO	54	5.0	-	3.99-6.01	-
**Previous Surgery**					
Yes	24	5.3	0.642	3.29-7.31	-
No	40	6.5	-	5.66-7.34	-
**Chemotherapy Regimen**					
Two drugs	55	6.3	0.002	5.03-7.57	0.960
Three drugs	9	5.8	-	3.17-8.43	-
**Cycle of Treatment**					
≤4	16	3.4	-	2.44-4.36	-
4-6	31	6.0	26.95	4.49-7.51	<0.01
>6	17	7.1	-	6.23-7.97	-
**No. of Metastatic Organs**					
0	9	7.7	-	7.26-8.14	-
1-2	16	7.5	34.336	6.33-8.67	<0.01
>2	39	4.5	-	3.90-5.10	-
**Lymph Node Metastasis**					
Present	55	6.3	0.002	5.03-7.57	0.960
Absent	9	5.8	-	3.17-8.43	-
**Syndrome Differentiation of TCM**					
Pyretic toxicity	29	5.9	-	4.42-7.38	-
Deficiency of spleen	7	3.3	17.528	2.22-4.38	0.01
Hygrosis	12	4.5	-	3.16-5.84	-
Blood stasis	16	7.1	-	6.12-8.08	-

**Table 3:** Multivariate analysis of PFS in the treatment group.

**Factors**	**B**	**S.E.**	**Wald**	***P*-Value**	**Exp (β) (95%CI)**
Lymph node metastasis	2.993	0.817	13.426	<0.001	19.953
**Cycle of Treatment**					
4-6 *vs.*<4	-1.050	0.484	4.709	0.030	0.350
>6 *vs.*<4	-1.682	0.606	7.696	0.006	0.186
**No. of Metastatic Organs**					
0 *vs.*>3	-1.244	0.617	4.064	0.044	0.288
1-2 *vs*.>3	-1.618	0.471	11.782	0.01	0.198

**Table 4:** Quality of life between two groups.

**Symptom**	**-**	**Mean**	**Median**	**Z**	***p***
Stomachache	Treatment group	15.97±2.63	11.11	-0.838	0.032
	Control group	16.92±2.31	11.11		
Body pain	Treatment group	19.14±2.37	16.67	-2.173	0.030
	Control group	27.44±2.65	33.33		
Reflux symptom	Treatment group	25.83±2.74	22.22	-2.148	0.032
	Control group	35.04±3.14	33.33		
Dietary restriction	Treatment group	30.03±2.53	33.33	-1.263	0.206
	Control group	27.01±2.73	33.33		
Anxious	Treatment group	34.03±2.94	33.33	-0.339	0.735
	Control group	32.86±3.54	33.33		
Psychology	Treatment group	20.31±2.53	33.33	-0.260	0.795
	Control group	22.05±2.86	33.33		
Holistic health	Treatment group	16.15±2.35	00.00	-0.449	0.617
	Control group	15.38±2.64	00.00		
Taste change	Treatment group	16.67±2.67	00.00	-0.08	0.994
	Control group	16.41±2.33	00.00		

**Table 5:** Toxic profiles of patients.

**Toxicities**	**Treatment Group (n = 64)**		**Control Group (n = 65)**	
	**Any Grade**	**Grade ≥3**	**Any Grade**	**Grade ≥3**
Leucopenia	26 (40.6%)	1 (1.6%)	32 (49.2%)	4 (6.2%)
Anemia	16 (25%)	1 (1.6%)	18 (27.7%)	3 (4.6%)
Thrombocytopenia	17 (26.6%)	0	21 (32.3%)	0
Nausea/Vomiting	23 (36.0%)	0	34 (52.3%)	2 (3.1)
Pigmentation	19 (29.7%)	0	31 (47.7%)	3 (4.6%)
Hypertension	7 (10.9%)	0	13 (20%)	1 (1.5%)
Hand-foot syndrome	16 (25.0%)	0	19 (29.2%)	1 (1.5%)
Oral mucositis	8 (12.5%)	1 (1.6%)	7 (10.8%)	0
Diarrhea	14 (21.9%)	1 (1.6%)	19 (29.2%)	3 (4.6%)
Hepatorenal dysfunction	20 (31.3%)	0	28 (43.1%)	5 (7.7%)

## Data Availability

The data and supportive information are available within the article.

## LIST OF ABBREVIATIONS

AEs: Adverse Events
ECOG: Eastern Cooperative Oncology Group
FJQR: “Fuzheng Jiedu Quyu Method” Recipe
GC: Gastric Cancer
HER-2: Human Epidermal Growth Factor Receptor 2
ORR: Overall Remission Rate
OS: Overall Survival
PFS: Progression-free Survival
PS: Performance Status
QoL: Quality of Life
TCM: Traditional Chinese Medicine
